# Male involvement in birth preparedness and complication readiness for emergency referral at Sodo town of Wolaita zone, South Ethiopia: a cross sectional study

**DOI:** 10.1186/s12884-020-2758-9

**Published:** 2020-01-30

**Authors:** Kebreab Paulos, Nefsu Awoke, Bazie Mekonnen, Aseb Arba

**Affiliations:** 1 Department of Midwifery, College of Health and Medical Sciences, Wolaita Sodo University, Wolaita Sodo, Ethiopia; 2 Department of Nursing, College of Health Science and Medicine, Wolaita Sodo University, Wolaita Sodo, Ethiopia; 3 Department of Midwifery, College of Health and Medical Sciences, Addis Ababa University, Addis Ababa, Ethiopia

**Keywords:** Male involvement, Birth, Obstetric complications, Emergency referral, Wolaita zone, Sodo town

## Abstract

**Background:**

Preventable maternal mortality remains a huge burden more especially in sub-Saharan Africa. The involvement of male partner during pregnancy and its complication helps an expectant mother to make timely decisions to avoid delays that brings about complications that could result in morbidity or mortality.

**Methods:**

Institution based cross sectional study was conducted in 2017, at Sodo Town of Wolaita Zone among mothers who came to hospital and admitted to MCH department due to emergency obstetric referral. Data were collected using pre-tested and structured questionnaire. The collected data entered by Epi data, cleaned and analyzed by using SPSS for windows version 23.0. A descriptive analysis was done using frequency, mean, quartile and standard deviation. Bivariate and multivariable logistic regression was carried out to identify the associated factors. Level of statistical significance was declared at *p* value < 0.05. Finally the results of Bivariate and multivariable logistic regression analysis were presented in crude and adjusted odds ratio with 95% confidence intervals.

**Result:**

Data were obtained from 233 women, with a response rate of 100%. The prevalence of male partner’s involvement in birth preparedness and complication redness for emergency referral in this study was 30.9%. After adjusting for the effect of confounding variables using multivariable logistic regression, variables like distance of health facility (AOR = 0.29, 95%CI = 0.12, 0.72), having ANC follow-up (AOR = 2.9, 95%CI = 1.52–5.51) and experience of obstetric complication (AOR = 1.79, 95%CI = 1.06–3.04) have statistically significant association with male partner’s involvement in birth preparedness and complication readiness for obstetric referral.

**Conclusion:**

In general, male partner’s involvement in birth preparedness and complication readiness for obstetric referral in the study area was low. Antenatal care attending and experiencing of obstetric complication were factors determining male partner’s involvement in complication readiness. Health care professionals should involve male partners to attend ANC clinic at each stage and arrange special antenatal care conferences which may increase awareness and practice about complication readiness and plan.

## Background

Maternity carries risk on life of women due to obstetric and emergent complication. Maternal deaths in developing countries share almost all of maternal death of the world, which accounts for more than a million of all deaths [1]. World Health Organization (WHO) reported that maternal death in developing countries accounted for 286,000 of all maternal deaths as a consequence of preventable complications. These deaths are caused from prenatal period complications [2].

In our country Ethiopia, the ratio of maternal mortality (MMR) is estimated to be 412 deaths per 100,000 live births according to the report from Ethiopian Demographic Health Survey (EDHS) [3]. In our country Ethiopia, the ratio of maternal mortality (MMR) is estimated to be 412 deaths per 100,000 live births according to the report from Ethiopian Demographic Health Survey (EDHS) [3]. It was declined from 1400 in 1990 to 412 in 2015 by 71% which was subsequently near the objective for MDG 5 (75%) [4].

Regardless of these general accomplishments in the health related MDGs in Ethiopia, there were additionally difficulties that would likewise be worries during the time of the SDGs. Government expense on health is 4.9% which is still underneath the Abuja responsibility to burn through 15% of the administration spending plan on health. Per capita health utilization was underneath 2001 WHO recommendation to be US$35 however it was US$26.7 in 2014 [4]. To tackle this Ethiopia has adapted the current global development agenda (SDGs) which has 17 goals, with Goal 3 focusing on health (ensure healthy lives and promote well-being for all at all ages) [5].

A main strategy to reduce maternal mortality and morbidity from complications is equipping family with awareness of birth plan that include birth-preparedness and complication readiness procedures specially for pregnant women, their husbands and their families [6]. Birth preparedness and complication readiness (BP/CR) is a wide-ranging and inclusive package aimed at encouraging timely access to skilled maternal and neonatal services, promotes active preparation and decision making for delivery by pregnant women and their families [7].

The most important components of birth plan package include recognition of danger sign, a plan for birth attendant, a plan for place of delivery, saving money for transport or other costs in case the need arise. In sub-Saharan Africa, pregnancy and childbirth remain to be viewed merely as women issues [8]. A male companion during perinatal care is unusual and rare in many communities, it is unthinkable to find male partners accompanying a woman to the labor room during delivering [9].

Nevertheless, men have social and economic power, especially in Africa, and have significant control over their partner. They decide the timing and condition of sexual relation, family size, whether their partner will utilize available maternal health care [10]. Hence this condition makes male partners participation critical if improvement in maternal health and reduction of maternal morbidity and mortality is to be realized. Strategies for enhancing involvement of men in maternal health services should aim at raising their awareness about emergency obstetrics conditions, and engaging them in birth preparedness and complication readiness [11].

Maternal mortality and morbidity associated to pregnancy and childbirth could be prevented if women and their families identify when and where to seek help, have access to the healthcare system during pregnancy, childbirth and the postpartum period and subsequently receive care from skilled provide [12].

Lack of husband participation in birth preparedness plans and delays in care seeking for obstetric emergencies are main contributing factor of maternal death. Male involvement is essential for improving birth preparedness because of patriarchy which agree to men to control women’s access to and utilization of maternal health care and safe motherhood programs which may be affected by male partner participation because husbands were the most influential decision-maker and as the key member of the family [13].

Despite the great ability of Birth Preparedness and Complication Readiness in reducing the maternal and newborn deaths, its importance is not well known in most of sub-Saharan Africa. Therefore this study aimed to assess male involvement in birth preparedness and complication readiness for emergency referral.

## Methods

### Study area

The study was conducted in Sodo Christian General Hospital (SCGH) and Wolaita Sodo University Teaching and Referral Hospital (WSUTRH) which is located in Sodo town, Wolaita Zone. Wolaita Sodo is main town of Wolaita Zone, Southern Nations, Nationalities, and Peoples Region (SNNPR) of Ethiopia. Wolaita Sodo is located 332 km south of Addis Ababa and 122 km south of Hawassa. Sodo Christian General Hospital is only private hospital in Sodo Town and WSUTRH, former Otona Hospital is recently held by Wolaita Sodo University. These hospitals serve population of Wolaita Zone and surrounding zones and regions of Ethiopia as referral hospital.

### Study design and period

A cross-sectional study design was conducted at Sodo Christian general hospital and Wolaita Sodo university teaching and referral hospital maternity ward from July 01 to October 30, 2017.

### Study population

All mothers who were admitted at maternity unit of two hospitals due to obstetric emergency were source population and mothers who are admitted to maternity unit due to obstetric emergency during the period of data collection were considered as study population. The study inclusion criteria were having been admitted to the hospital as an emergency referral in antenatal, labor or the postpartum period and willingness to consent for participation in the study. Participants were followed up to their discharge from hospitals or death.

### Sample size and sampling technique

Sample size was determined by using single population proportion formula based on the following assumptions: 95% confidence level, prevalence of male involvement in birth preparedness and complication readiness is 60.4% from previous study [14], and a 5% margin of error. The Calculated sample size was *n* = 368.

The last quarter (3 months) data of the two hospitals show that in total of 500 (292 in WSUTRH and 208 SCGH) mothers were admitted to maternity unit due to obstetric complications. Since total population of this study less than 10,000 we used correction formula.

$$ n=\frac{n}{1+\frac{n}{N}}=\frac{368}{1+\frac{368}{500}} $$

Expecting a 10% non-response rate the final sample size calculated was 233.

The total sample size was allocated proportionally to each hospital by reviewing the numbers of mothers who were admitted to maternity unit due to obstetric complications in the last quarter of 2016. Systematic random sampling technique was utilized to approach the study participant who fulfills the inclusion criteria. The lists of mothers were obtained from admission registry of maternity unit, and this was used to draw sample of the study participant. Calculated Kth value is 2 and first random start 1 chosen by lottery method and data were collected every 2nd interval.

### Data collection tool

Interviewer administered questionnaires were conducted for the quantitative study. The questionnaire was adapted from the survey tools developed by JHPIEGO Maternal and Neonatal Health Program [15]. It is divided into four parts. The first section inquired about personal data, including age, occupation, ethnicity, religion and educational level. The second part elicited information about Knowledge of obstetric complication and Sours of information. The third section assessed birth preparedness and complication readiness and health seeking behaviors.

Male partner involvement: refers to its involvement in joint decisions on where to attend ANC, where to deliver, save money for emergency, knowing and deciding earlier on where to go during emergency case.

### Data collection process

Four experienced female data collectors were collected the data after thorough training on the objective of the study and the questionnaire. Two degree holder health professionals supervised the data collectors. Data collectors and supervisors were trained for 5 days by using training manual prepared for this purpose. Data collection was conducted after women managed for the emergency case and willingness to respond to the questions.

### Data analysis

The collected data coded and entered by Epi data version 3.1 and cleaning and analyzed by SPSS version 23.0. A descriptive analysis was done using frequency, mean, quartile and standard deviation. Bivariate logistic regression was carried out to identify the associated factors. All variables with *p* value ≤0.25 were taken into consideration in the multivariable model to control for all possible confounders and the variables were selected by enter method to see the efect of each variable on the outcome variable. Multicollinearity tests were carried out to see the correlation between independent variables using standard error and one of the independent variable was dropped for those with standard error of > 2. Finally the results of multivariable logistic regression analysis were presented in crude and adjusted odds ratio with 95% confidence intervals. Level of statistical significance was declared at *p* value < 0.05.

### Data quality control

To maintain the validity of the data collection tool the questionnaire was first translated to Amharic then to Wolaitigna and retranslated to English after data collection to maintain consistency by native language translator. Training on data collection and procedure was provided to data collectors and supervisors. Pretest was conducted on 5% of sample size in Areka town Dubo hospital.

### Ethical considerations

Ethical clearance letter was obtained from Addis Ababa University Department of Nursing and Midwifery research committee and College of Health Sciences Institutional Review Board. Written permission was requested from Wolaita Sodo zone health bureau. Data collectors given consent to collect right data from participants and to respect rights of participants. Moreover, all the study participants were informed verbally about the purpose and benefit of the study along with their right to refuse and verbal consent was obtained. Furthermore, the study participants are reassured for an attainment of confidentiality and written consent information is also taken.

## Results

### Socio-demographic characteristics of respondents and male partner

A total of 233 Married women who had come to hospital due to emergency obstetric referral were interviewed with response rate of 100%. Majority of the women were in the age group of 25 and above (72.5%) with mean age of 26.7 ± 4.8 standard deviation, while most of male partners were in the age group of 30 and above years of age (73%) with mean age of 31.9 ± 4.9 standard deviation. Regarding educational level (33.5%) women and 36.5% of male partners had primary level of education. Most of the women were housewives (47.5%) whereas (38.3%) of male partners were Gov. Organization employed (Table 1) both women and male partners were similar in their religion.

**Table 1:** Socio-demographic characteristics of the Women and Male partners in Wolaita zone Sodo town, 2017

Variables (*n* = 233)	Category	n (%)
Women age (Years)	< 25	64 (27.1)
	≥25	169 (72.5)
Male partners Age (Years)	< 30	63 (27)
	≥ 30	170 (73)
Women Educational Level	No Formal Education	50 (21.5)
	Primary (1–8)	78 (33.5)
	Secondary (9–12)	53 (22.7)
	Tertiary (above 12)	52 (22.3)
Male partners Educational Level	No Formal Education	19 (8.2)
	Primary (1–8)	85 (36.5)
	Secondary (9–12)	50 (21.5)
	Tertiary (above 12)	79 (33.9)
Women Occupation	Gov.Org.Employed	45 (19.3)
	NGO or Private Org. Employed	3 (1.3)
	Merchant	49 (21)
	Daily Laborer	7 (3)
	Housewife	111 (47.5)
	Other	18 (7.7)
Male partners Occupation	Gov.Org.Employed	66 (28.3)
	NGO or Private Org. Employed	14 (6)
	Merchant	63 (27)
	Daily Laborer	38 (16.3)
	Farmer	51 (21.9)
	Other	1 (0.4)

### Birth preparedness and complication readiness plan

Majority 214(91.8%) of the respondents had a plan for delivery. Ten (4.3%) of the women delivered at home previously from those women 4(1.7%) mentioned distance from health facility is too far as reason for home delivery. Midwifes/nurse assisted 119(51.1%) of women delivery. Twenty one (9%) of women experienced problem during pregnancy of them 13(5.6%) experienced bleeding during pregnancy, 16(6.9%) seek health care due to the problem (Table 2).

**Table 2:** Birth preparedness and health seeking behaviors at Sodo town in Wolaita zone, 2017

Variables (*n* = 233)	Category	n (%)
Made a plan for deliver	Yes	214 (91.8)
	No	19 (8.2)
Place of previous delivery	Health facility	223 (95.7)
	Home	10 (4.3)
Reason for home delivery(*n* = 10)	Far distance from health facility	4 (1.7)
	No transportation	3 (1.3)
	Cost too much	2 (0.9)
	Delay to seek care	1 (0.4)
Delivery assisted by	Midwife or Nurse	119 (51.1)
	Doctor	100 (42.9)
	HEW	7 (3)
	Others	7 (3)
Experienced problem during pregnancy	Yes	21 (9)
	No	212 (91)
Problems experienced	Bleedings	13 (5.6)
	Loss of consciousness	3 (1.3)
	Severe headache	2 (0.9)
	Other	5 (2.1)
Seek health care during problem and complications	Yes	16 (6.9)
	No	5 (2.1)

### Level of male partners involvement on decision for antenatal care follow-up

**Fig. 1:**
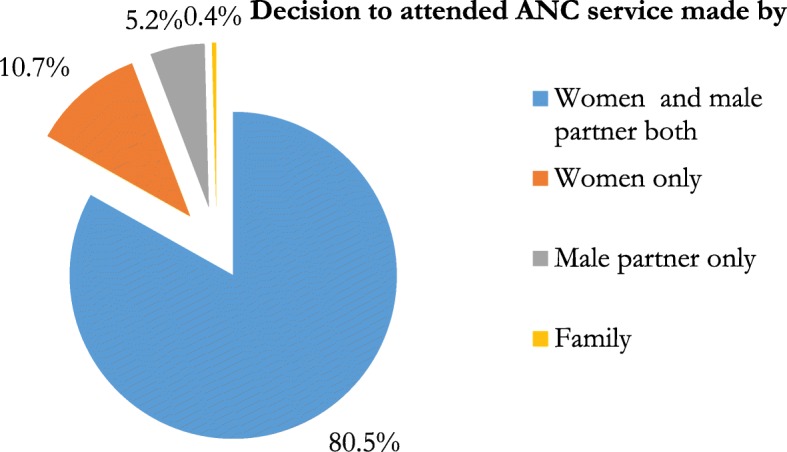
Decision to attended ANC service of the Women and Male partners at Sodo town in Wolaita zone, 2017

Majority 225(96.6%) of the women received ANC at least once and the rest 8(3.4%) do not attended ANC service. One hundred eight seven (80.5%) of women and their male partner both decided to follow ANC (Fig. 1).

### Reason for referral male partners accompanied to place of delivery

Among the reason for referral 69(29.6%) were referred due to obstructed or prolonged labor followed by 34(14.6%) fetal distress, 27(11.6%) Malpresentation, 20(8.6%) Previous caesarean section, 16(6.9%) Multiple pregnancy, 16(6.9%) Postpartum hemorrhage, 16(6.9%) Anemia, 14(6%) hemorrhage, 13(5.6%) Poor obstetric history, 10(4.3%) Ruptured uterus, 9(3.9%) Eclampsia, 7(3%) Preeclampsia, and 5(2.1%) Preterm labour. From the women referred to hospital majority 189(81.1%) reached hospital by ambulance. Among women referred to hospital 75(32.2%) were accompanied to deliver place by male partner and majority 156(67%) of women were accompanied by other member of the family member. Majority 164(70.4%) of the women get health facility in distance of greater than 5 km (Table 3).

**Table 3:** Male partner involvement in decision making for health care seeking at Sodo town in Wolaita zone, 2017

Variables (*n* = 233)	Category	n(%)
Means of transportation	Ambulance	189 (81.1)
	Privet car	23 (9.9)
	Taxi/bus	21 (0.9)
Who accompanied to place of delivery	Male partner	75 (32.2)
	Other member of the family	156 (67)
	TBA	2 (0.9)
Distance of health facility from home	≥5 km	164 (70.4)
	< 5 km	69 (29.6)

### Factors associated with male partner involvement during birth preparedness and complication readiness for obstetric referral

There were seven variables in binary logistic regression which had a *p*-value of ≤0.25 and became a candidate for multiple logistic regressions. After adjusting for the effect of confounding variables using Multiple logistic regression, three variables like distance of health facility (AOR = 0.29, 95%CI = 0.12, 0.72), having ANC follow-up (AOR = 2.9, 95%CI = 1.52–5.51) and experience of obstetric complication (AOR = 1.79, 95%CI = 1.06–3.04) have statistically significant association with male partner’s involvement in birth preparedness and complication readiness for obstetric referral (Table 4).

**Table 4:** Factors associated with male involvement during birth preparedness and complication readiness for obstetric referral at Sodo town in Wolaita zone, 2017

Variables	Male involvement		COR (95%CI)	AOR (95% CI)	*P*-value
	Yes	No			
Women Educational					
Primary and below	26 (20.3%)	102 (79.68%)	0.23 (0.09–0.64%)	0.94 (0.24–3.61)	.924
Secondary and above	55 (52.38%)	50 (47.61%)	1.00	1.00	
Male partner education					
Primary and below	89 (51.44%)	84 (48.55%)	1.60 (1.07–2.61)	1.09 (0.43–2.74)	.858
Secondary and above	24 (40%)	36 (60%)	1.00	1.00	
Women Occupation					
Formal employment	48 (85.71%)	8 (14.28%)	1.86 (0.51–7.53)	2.48 (0.57–10.83)	.227
Informal employment	135 (76.3%)	42 (23.72%)	1.00	1.00	
Male partner occupation					
Formal employment	13 (16.3%)	67 (83.75%)	0.09 (0.01–0.73)	0.09 (0.01–1.04)	.054
Informal employment	105 (68.6%)	48 (31.37%)	1.00	1.00	
Distance of health facility					
Less than 5 km	10 (14.5%)	59 (85.5%)	0.23 (0.11–0.56)	0.29 (0.12–0.72)	.008
More than 5 km	69 (15.76%)	95 (84.2%)	1.00	1.00	
ANC at least once					
Yes	105 (46.6%)	120 (53.33%)	2.62 (1.52–4.74)	2.9 (1.52–5.51)	.001
No	2 (25%)	6 (75%)	1.00	1.00	
Experience of complication					
Yes	1 (4.76%)	20 (95.2%)	1.63 (1.07–2.64)	1.79 (1.06–3.04)	0.030
No	16 (7.54%)	196 (92.5%)	1.00	1.00	

Statistically significant at *p* < 0.05

## Discussion

The aim of this study was to assess male partners’ involvement in in birth preparedness and complication readiness for obstetric referral of a spouse and its associated factors among women admitted at MCH department due to obstetric referral at Sodo town in Wolaita zone, Southern Ethiopia.

In this study prevalence of male partners’ involvements’ in birth preparedness and complication readiness for obstetric referral was 30.9%. Women who had any experience of pregnancy complications were 1.79 times and those who received ANC follow up at list one time were 2.9 times more likely to involve male in birth preparedness and complication readiness for obstetric referral whereas women’s who walked less than 5 km distance to the nearest health facility had lower odds of male partners involvement in birth preparedness and complication readiness for obstetric referral compared to those who walked more than 5 km.

Having ANC follow-up of women at list once was significant predictor for male partners’ involvement in birth preparedness and complication readiness for obstetric referral. Respondent who had ANC follow-up at list once were 2.9 times more likely male involve in birth preparedness and complication readiness for obstetric referral than those had no ANC follow up. The finding was consistent with other study done in Haiti, Ambo and Addis Ababa [9, 16, 17]. Having ANC follow-up at list once could influence male partner’s involvement in birth preparedness and complication readiness for obstetric referral.

Respondent who walk more than 5 km from home to nearest health facility to have obstetric care, delivery and PNC services were 0.28 times highly accompanied by male partners compared to those who walk less than 5 km. The finding was consistent with other study done Addis Ababa [16].

Our study also showed that women who had any experience of pregnancy complications were 1.79 times more likely to be accompanied by male partner in birth preparedness and complication readiness for obstetric referral than those who didn’t have any signs of complication. The finding was consistent with other study done in Uganda [18]. This may be due to spouses might have been worried more than those whose spouses had not experienced any signs of pregnancy complications.

## Conclusions

In general, male partner’s involvement in birth preparedness and complication readiness for obstetric referral in the Sodo Town, Wolaita Zone was low. Antenatal care attending and experiencing of obstetric complication were taking major part in determining male partner’s involvement in complication readiness. Health care professionals should involve male partners to attend ANC clinic at each stage and arrange special antenatal care conferences which may increase awareness and practice about complication readiness and plan.

## Data Availability

The datasets used and/or analyzed during the current study are available from the corresponding author on reasonable request.

## Abbreviations

ANC: Antenatal Care
AOR: Adjusted odds ratio
EDHS: Ethiopian Demographic Health Survey
FMOH: Federal Ministry of Health
HEWS: Health Extension Workers
JHPIEGO: John Hopkins Program for International Education in Gynecology Obstetrics
MCH: Maternal and Child Health
MMR: Maternal Mortality Ratio
TBA: Traditional Birth Attendant
